# Soil origin and plant genotype structure distinct microbiome compartments in the model legume *Medicago truncatula*

**DOI:** 10.1186/s40168-020-00915-9

**Published:** 2020-09-28

**Authors:** Shawn P. Brown, Michael A. Grillo, Justin C. Podowski, Katy D. Heath

**Affiliations:** 1 Department of Plant Biology, University of Illinois, 505 S. Goodwin Ave, Urbana, IL 61801 USA; 2 Carl R. Woese Institute for Genomic Biology, University of Illinois, 1206 W. Gregory Dr, Urbana, IL 61801 USA; 3 Department of Biological Sciences, The University of Memphis, 3774 Walker Ave, Memphis, TN 38152 USA; 4 Center for Biodiversity Research, The University of Memphis, 3774 Walker Ave, Memphis, TN 38152 USA; 5 Department of Biology, Loyola University Chicago, 1032 W. Sheridan Rd, Chicago, IL 60618 USA; 6 Department of Geophysical Sciences, University of Chicago, 5734 S Ellis Ave, Chicago, IL 60637 USA

**Keywords:** Common garden, Evolution, Genetic variation, Mutualism, Nodule microbiome

## Abstract

**Background:**

Understanding the genetic and environmental factors that structure plant microbiomes is necessary for leveraging these interactions to address critical needs in agriculture, conservation, and sustainability. Legumes, which form root nodule symbioses with nitrogen-fixing rhizobia, have served as model plants for understanding the genetics and evolution of beneficial plant-microbe interactions for decades, and thus have added value as models of plant-microbiome interactions. Here we use a common garden experiment with 16S rRNA gene amplicon and shotgun metagenomic sequencing to study the drivers of microbiome diversity and composition in three genotypes of the model legume *Medicago truncatula* grown in two native soil communities.

**Results:**

Bacterial diversity decreased between external (rhizosphere) and internal plant compartments (root endosphere, nodule endosphere, and leaf endosphere). Community composition was shaped by strong compartment × soil origin and compartment × plant genotype interactions, driven by significant soil origin effects in the rhizosphere and significant plant genotype effects in the root endosphere. Nevertheless, all compartments were dominated by *Ensifer*, the genus of rhizobia that forms root nodule symbiosis with *M. truncatula*, and additional shotgun metagenomic sequencing suggests that the nodulating *Ensifer* were not genetically distinguishable from those elsewhere in the plant. We also identify a handful of OTUs that are common in nodule tissues, which are likely colonized from the root endosphere.

**Conclusions:**

Our results demonstrate strong host filtering effects, with rhizospheres driven by soil origin and internal plant compartments driven by host genetics, and identify several key nodule-inhabiting taxa that coexist with rhizobia in the native range. Our results set the stage for future functional genetic experiments aimed at expanding our pairwise understanding of legume-rhizobium symbiosis toward a more mechanistic understanding of plant microbiomes.

Video Abstract

## Background

Plants grow in close co-association with a striking diversity of microorganisms [1]. These microbes, including bacteria, archaea, fungi, and protists, can inhabit every conceivable plant organ and tissue as either epiphytes or endophytes. A rapidly growing body of literature has documented the influence that the microbiome can have on critical plant traits including disease resistance [2–5], nutrient acquisition and growth [6–9], abiotic stress tolerance [10, 11], and flowering phenology [12, 13]. Thus, the microbiome can be viewed as an extended phenotype of the plant genome that can enhance the ability of plants to cope with environmental stressors [1, 10, 14–16]. A fuller understanding of plant microbiomes is critical for improvements in environmental sustainability [17], agriculture [18], and conservation [19]. To leverage microbiomes to address critical needs, we must better understand the factors that structure microbial communities within and among plant hosts, building a predictive understanding of microbiome assembly.

The advent of modern sequencing technology has provided a renaissance for microbial ecology by allowing for rigorous characterization of microbial communities and their relationships with macrobial hosts [20]. Some authors have even suggested that the hologenome, comprised of the host’s genome and all genomic content of associated microbes, is a unit of biological organization driving ecological and evolutionary processes [21, 22]. Microbiomes are diverse and have been found to vary across plant species [23–25], within species among different genotypes [26–30], and among plants grown in different environments [29, 31–33]. Additionally, distinct compartments within a plant (e.g., phyllosphere, rhizosphere, endosphere) often vary in microbiome composition [1, 29, 33]. Thus, environmental and genetic factors working together largely determine plant microbiome assembly.

Soil communities can vary considerably in space and time, leading to variation in the microbial pool available for colonization [34, 35]. For roots, a two-step model for microbiome colonization has been proposed [1, 33], wherein root exudates initially drive a shift in community composition in the soil directly influenced by the root (i.e., the rhizosphere), followed by plant genetic factors that regulate entry inside the root (i.e., the root endosphere). The acquisition of the leaf microbiome (i.e., phyllosphere) is less well-understood, but likely depends on similar multi-level processes [36, 37]. In order to gain entry into the endosphere (interior of the leaves, roots, stem, etc.), microbes must overcome plant innate immunity [38]. The field of community genetics has long held that intraspecific genetic variation, and thus intraspecific evolution, can scale up to influence community and ecosystem-level processes [39]. In plants, substantial genetic variation is maintained for plant immune response machinery [40], and numerous other ecologically relevant phenotypes; thus, it is perhaps not surprising that host genotypes vary in microbiome composition [24, 28, 29, 33]. Yet many microbiome studies have focused on only a single genotype; therefore, studies using multiple genotypes and wild species are necessary to better resolve the role of host plant genotypes in structuring the microbiome, particularly since domesticated species may be inferior in their ability to regulate microbiomes when compared to wild relatives [41–43].

Leveraging existing knowledge in well-studied models for plant-microbe interactions can help us better understand the factors structuring plant microbiomes. Leguminous plants (Fabaceae) are one of the most diverse lineages (ca. 20,000 spp.), and legumes are the second most important crop family behind grasses [44]. For decades, legumes have served as important model systems for understanding the genetics, ecology, and evolution of plant-microbe interactions because they form intimate symbioses both with nodulating nitrogen-fixing bacteria (rhizobia) and with arbuscular mycorrhizal fungi (AMF). Decades of molecular genetic work have uncovered numerous genes that are required for symbiosis with these key symbionts [45–47], and a subset of these so-called “symbiosis genes” have been implicated in interactions between legumes and a diversity of microorganisms beyond rhizobia and AMF [48–50]. Zgadzaj et al. [51], for example, detected a shift in microbiome community composition in mutant *Lotus japonicus* that were defective in nodulation, compared to wild type plants. This line of inquiry suggests that core symbiosis genetic pathways can influence interactions with a broad range of microbes beyond the model symbiosis in which they were discovered, and thus that models for plant-microbe symbiosis have added value in understanding plant microbiomes.

Microbial communities can be strongly structured by plant compartments, and this is potentially the case for legume nodules. Nodules represent a truly distinct environment from the adjacent root endosphere by being (1) low in oxygen, which is necessary for nitrogen fixation [52, 53], (2) rich in both carbon and nitrogen [54], and (3) dominated by single microbial taxon (i.e., rhizobia). Thus the nodule may harbor microbes that are specialists of this unique environment; however, this hypothesis has not been tested. Limited evidence in AMF supports this notion by demonstrating that communities differ between the root and the nodule, but are more similar between nodules of different legume species, suggesting that there may indeed be nodule AMF specialists [55]. Furthermore, the origin of nodule microbial communities is uncertain; bacteria could migrate into nodules from within roots, or there could be directed colonization from the rhizosphere. Culturing initiatives have revealed a diverse group of non-rhizobial species housed in nodules, and several of these bacteria can act to increase nodulation as well as overall plant growth (reviewed by [56]). In fact, commercially available inocula often include both rhizobia and non-rhizobial strains for this reason [57]. The mechanisms for enhanced nodulation and/or plant performance are unknown, but likely involve microbe-microbe interactions which could manifest inside the nodule or in other plant compartments. Interestingly, non-rhizobial members of nodule microbiomes can even possess nodulation and/or nitrogen fixation genes [56]. A rigorous characterization of nodule microbiomes that is not limited by culture bias is necessary to determine if specialist taxa exist in this unique compartment, and potentially shed light on functional interactions and mechanisms.

The model legume *Medicago truncatula* (hereafter *Medicago*), a winter annual native to the Mediterranean basin [58], has been used extensively to discover the genetic pathways necessary for establishment and ongoing symbiotic trade in both the rhizobium and AMF symbiosis [45, 59–61]. Despite its prominence in plant-microbe interactions, however, no studies have presented a thorough characterization of the *Medicago* microbiome grown in native soil, so to date we have an incomplete picture of the microbiome of this species beyond its interaction with *Ensifer* rhizobia. Here we first grow three *Medicago* genotypes from natural populations in each of two native soils in a common garden experiment to ask: (1) To what extent do plant genotype and soil origin structure the microbiome? (2) Do plant compartments (rhizosphere, root endosphere, nodule endosphere, phyllosphere) harbor distinct microbiomes, and how are these affected by plant genotype and soil origin? (3) Are there specialist microbial taxa in the nodule, and how is this community assembled (i.e., from the rhizosphere or root endosphere)? Next we perform an additional inoculation experiment, adding one of four *Ensifer* strains to ask: (4) Does genetic variation in rhizobia influence the broader microbiome, and in what compartments? Finally, we use additional shotgun metagenomic sequencing of a subset of root, nodule, and leaf samples to explore the genetic variation in *Ensifer* bacteria throughout the plant.

## Results

### Sequencing results—16S rRNA amplicon sequencing

After all sequence quality control, OTU demarcations, and removal of rare OTUs, we retained 3180 OTUs for the plant genotype × soil experiment and 1650 OTUs for the rhizobium genotype experiment. A detailed sample × OTU table is provided in Table S1, and OTU abundances across the sampling design with associated taxonomy, biomarker results, and representative sequences are available in Table S2.

### Sequencing results—shotgun metagenomic sequencing

After quality control, on average 41 million paired end reads per sample were retained. After removing read pairs that mapped to the *Medicago* reference, root endosphere samples contained on average six million read pairs and nodule samples contained 34 million read pairs. On average, 25% of the remaining reads successfully mapped to the reference (*E. medicae* WSM419) in root endosphere samples, versus 90% in nodule samples. Root endophyte samples with a high percentage of reads mapped to *E. medicae* WSM419 produced more than 30 scaffolds ranging from 50 to 427 kb, while nodule samples regularly produced 40 or more scaffolds greater than 50 kb, and each nodule sample produced more than 15 scaffolds greater than 100 kb.

### Plant genotype × soil experiment

**Fig. 1:**
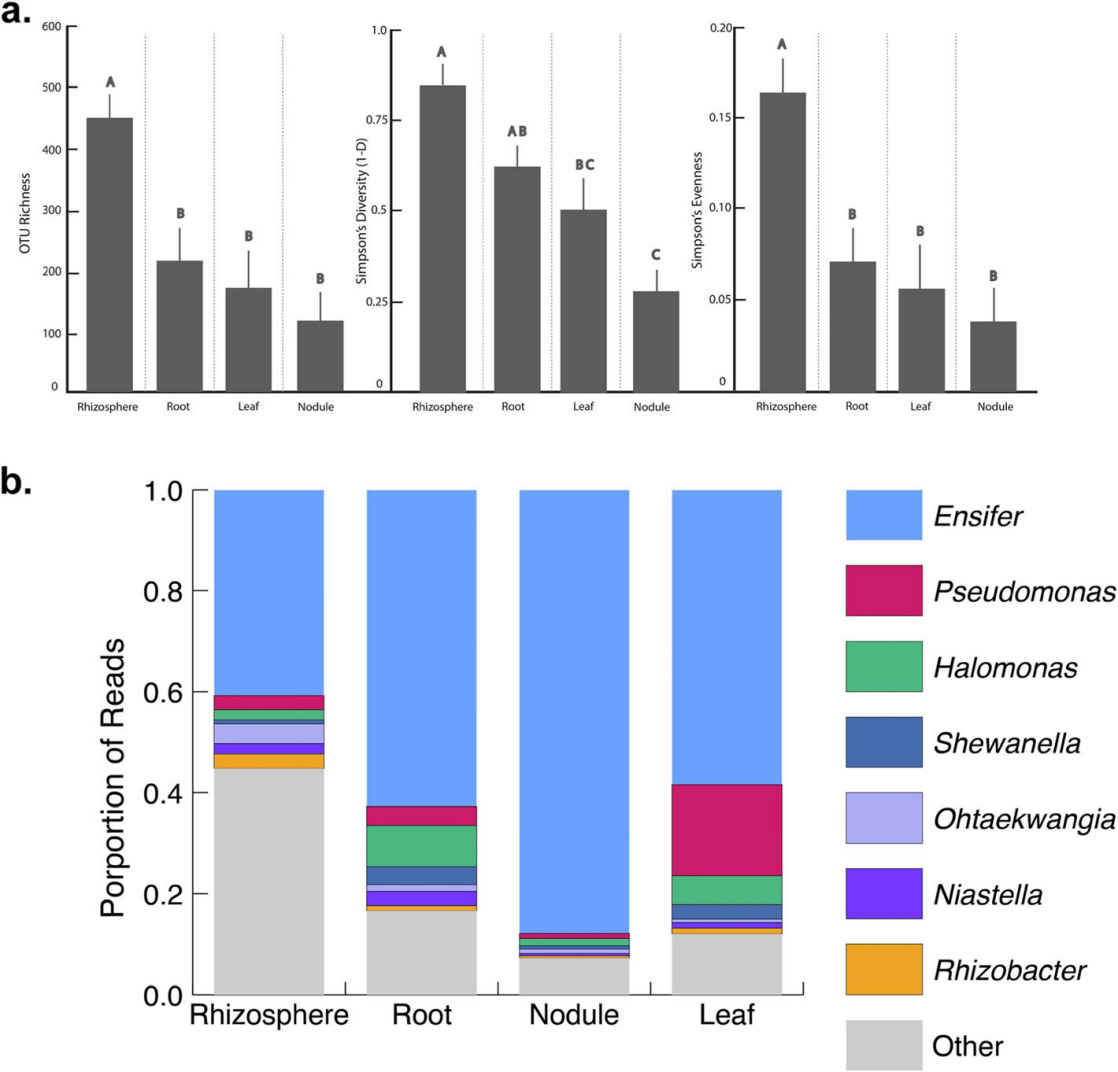
Bacterial diversity metrics (**a**) and abundance (mean ± SE) of the most abundant bacterial OTUs (**b**) for the four compartments studied. All estimates are based on iterative subsampling (1500 sequences per iteration at 1000 iterations). Letters represent significant differences (Tukey HSD) across compartments

Several results suggest that compartment was the major force structuring the microbiome of *Medicago* plants. First, PVCA indicated that interactions with compartment explained the largest amount of variance in bacterial community composition (compartment × soil source: 23%, and compartment × plant genotype: 6%; Table 1), indicating that soil origin and plant genotype both influenced community composition, but that these effects depended on compartment. Main effects of plant genotype, soil origin, compartment, and the soil origin × plant genotype interaction accounted for an additional 1.2–3.2% of the variation in bacterial composition each (~ 10.9% total; Table 1). ANOVAs on diversity estimators (diversity, evenness, and richness) indicated that compartment was the only significant effect (Table 1), with rhizospheres having the most rich, diverse, and even bacterial communities (Fig. 1a). We found no evidence for overall differences in diversity estimates among plant genotypes or between soil sources (Table 1).

**Table 1:** Results from modified principal variance component analysis (PVCA) enumerating the amount of community variation explained by soil origin (France of Corsica), plant genotype (G1, G27, G96), plant compartments (rhizosphere, root, nodule, leaf) and all possible interactions. Further, diversity estimator ANOVA results are presented across the same design. Complement of Simpson’s Diversity and Evenness were transformed using logit transformation and richness was transformed using Box-Cox functions prior to analysis

Test	Variance explained—PVCA (%)	Diversity (1-D)	Evenness (*E*_D_)	OTU richness (*S*_obs_)
Soil origin	3.207	*F* = 0.685, *P* = 0.411	*F* = 0.767, *P* = 0.384	*F* = 1.845, *P* = 0.178
Genotype	1.274	*F* = 0.054, *P* = 0.947	*F* = 1.356, *P* = 0.265	*F* = 0.0704, *P* = 0.498
Compartment	3.231	***F*** **= 14.151,** ***P*** **< 0.001**	***F*** **= 3.171,** ***P*** **= 0.029**	***F*** **= 39.509,** ***P*** **< 0.001**
Soil origin × genotype	3.222	*F* = 0.297, *P* = 0.753	*F* = 1.169, *P* = 0.316	*F* = 0.215, *P* = 0.807
Soil origin × compartment	23.098	*F* = 1.582, *P* = 0.202	*F* = 1.123, *P* = 0.346	*F* = 2.381, *P* = 0.077
Genotype × compartment	6.295	*F* = 0.884, *P* = 0.511	*F* = 0.702, *P* = 0.649	*F* = 1.379, *P* = 0.235
Soil origin × genotype × compartment	0.369	*F* = 0.634, *P* = 0.703	*F* = 0.496, *P* = 0.809	*F* = 0.488, *P* = 0.815
Residual	59.302

**Fig. 2:**
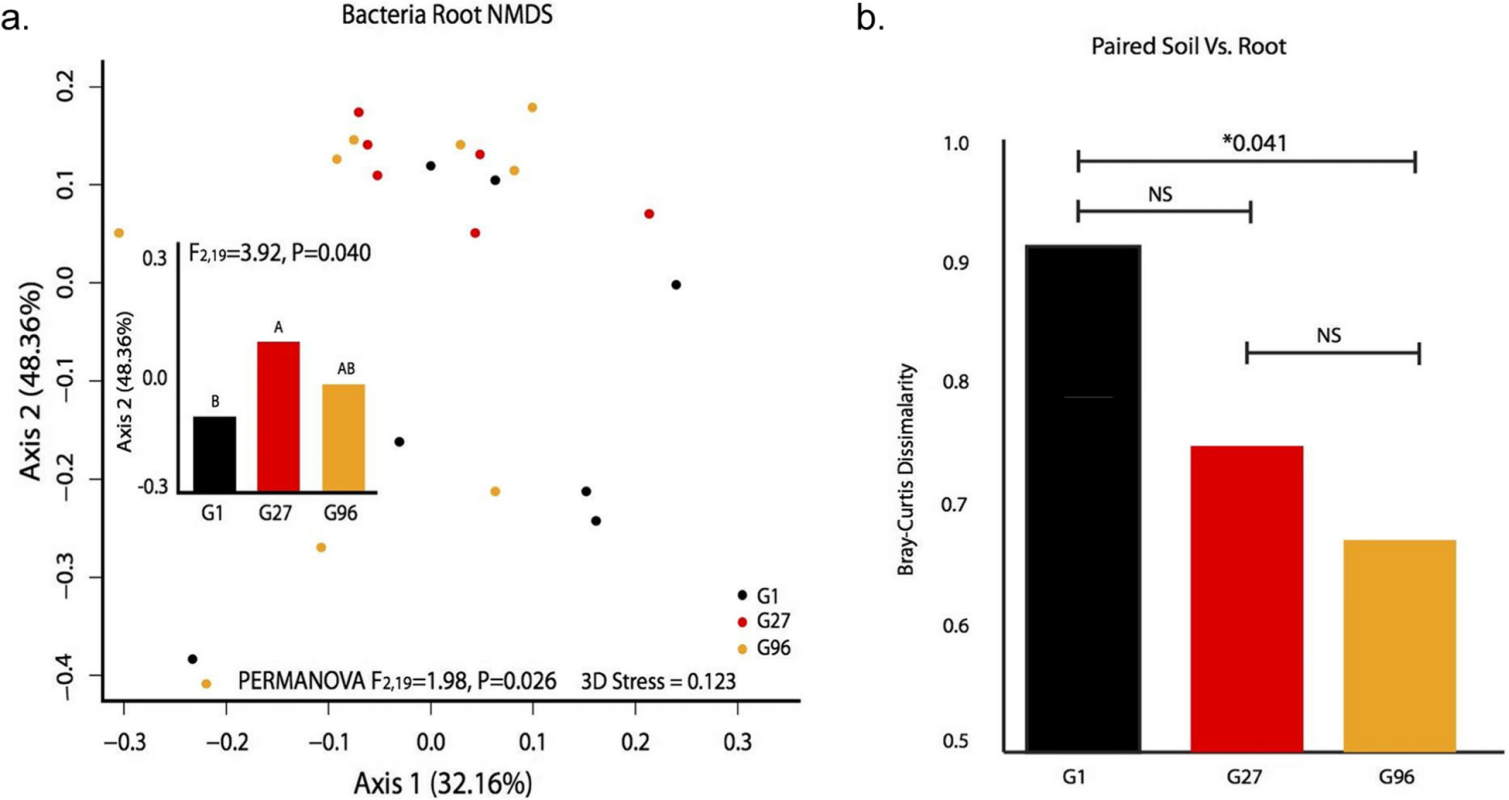
Bacterial communities in the root endosphere respond to plant genotype. **a** Nonmetric multidimensional scaling plot (Bray-Curtis) of bacterial root endophytes plotted by genotype, with insert showing Axis 2 loading scores (explains 48.36% of community variation) across genotypes (ANOVA). **b** Average bacteria Bray-Curtis dissimilarity values between paired rhizosphere and root endosphere samples (samples are paired by plant)

To unpack the interactions indicated in the PVCA, we next used PerMANOVAs to test for the effects of soil source and plant genotype on the composition of bacterial communities within each compartment separately. Rhizospheric communities were distinct between soils originating from mainland France vs. Corsica (Table 2, Figure S1, Figure S2), but this legacy of soil origin was lost in internal plant compartments (Table 2). Instead, bacterial communities within roots responded to plant genotype (Table 2, Fig. 2), though genotype was not significant for nodule or leaf compartments (Table 2).

**Table 2:** PerMANOVAs by compartment testing for the effects of soil origin (Corsica or France) and plant genotype on bacterial community composition. PerMANOVA tests are based on a Bray-Curtis dissimilarity matrix using an iterative subsampling of a depth of 1500 sequences (1000 iterations) per sample. Pseudo-*F* test statistics, degrees of freedom, and *P* values are presented, and significant tests are displayed in bold and in italics

Test	Soil origin Pseudo-*F*_df_	Soil origin *P* value	Genotype Pseudo-*F*_df_	Genotype *P* value
Rhizosphere	***F***_***1,28***_ ***= 4.838***	***0.001***	*F*_2,27_ = 0.762	0.679
Root	*F*_1,28_ = 1.006	0.374	***F***_***2,27***_ ***= 1.982***	***0.026***
Nodule	*F*_1,27_ = 1.057	0.330	*F*_2,67_ = 0.506	0.941
Leaf	*F*_1,28_ = 0.501	0.801	*F*_2,27_ = 1.052	0.411

**Fig. 3:**
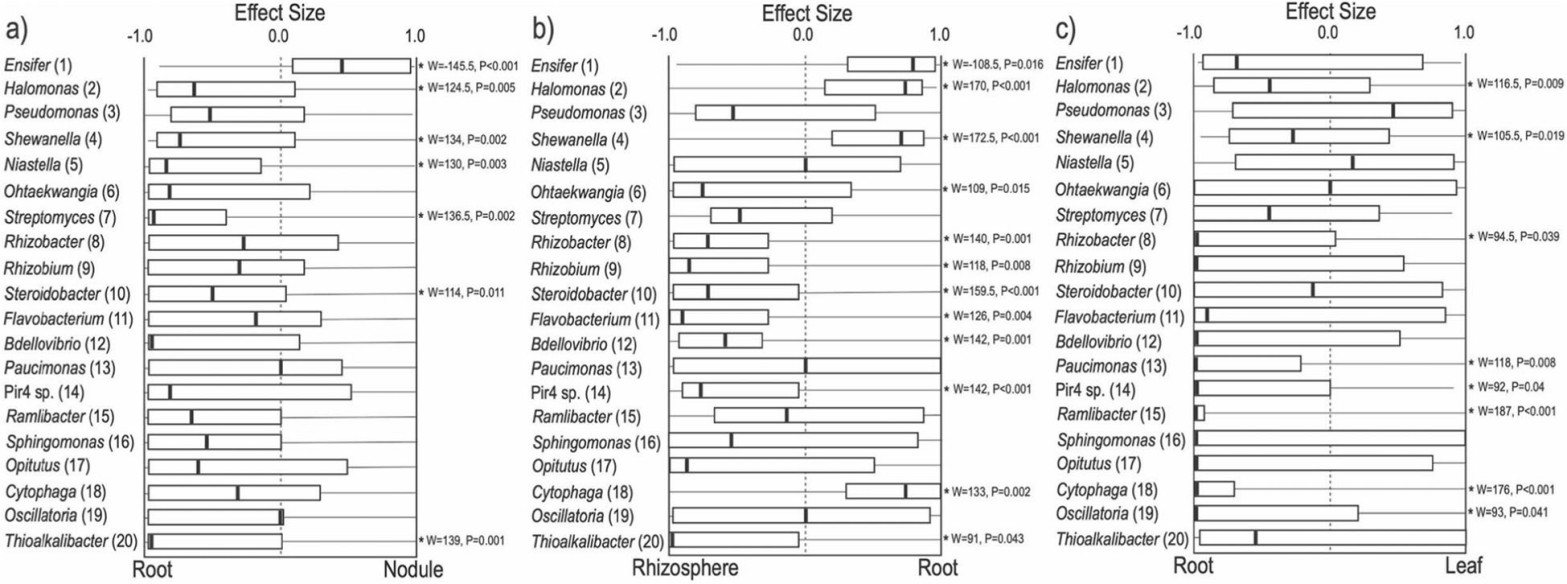
Paired effect size analysis of the 20 most abundant OTUs comparing relative abundances of each OTU within the same plant between root and nodule (**a**), rhizosphere and root (**b**), and root and leaf (**c**) compartments. Bacterial genera are on the left and OTU number presented parenthetically. Presented are quintiles (minimum, 25%, median, 75%, maximum) of paired effect size [e.g., nodule − root/(nodule + root)] where a value of 1.0 indicates this OTU is only found in the nodules where as a value of − 1.0 means the genus was only found in the Root. Tests for significant enrichment of OTUs between compartments using Wilcoxon signed-rank tests are presented where significant and indicated with tests statistics and *p* values

Bacterial communities were quite distinct across the four compartments (Table 1; Fig. 1b); only 327 (10%) of OTUs were shared across all compartments (Figure S1). The genus *Ensifer*, which contains the primary N-fixing rhizobia that form root nodule symbiosis with *Medicago*, was the most abundant in all compartments, though it reached higher abundances in internal plant compartments (particularly in the nodule, unsurprisingly, where it comprised ~ 85% of reads; Fig. 1b). Consistent with the diversity results, the rhizosphere compartment had the most (772) unique OTUs (Figure S1), and LEfSe analyses identified 313 biomarker OTUs for the rhizosphere, of wide-ranging taxonomic identities (see Table S2), some of which were common (Fig. 3b). Prominent shifts occurred in the abundances of other OTUs across internal plant compartments, including *Halomonas* (increased in root and leaf endospheres compared to nodules; Fig. 3a) and *Pseudomonas* (increased in leaves relative to root and nodule; Fig. 3c). Our LEfSe analyses identified 10 biomarker OTUs for leaves, dominated by *Pseudomonas*, *Niastella*, and the cyanobacteria *Phormidium* and 12 biomarker OTUs for roots dominated by *Thioalkalibacter*, *Neorhizobium*, and *Ohtaekwangia*, plus one OTU (*Ensifer*) for nodules (Table S2).

Examination of nodule core communities identified 15 OTUs that were found in > 50% of nodule samples, including some of the most overall abundant OTUs (Table S3). Common nodule taxa included other putative N-fixing or plant growth promoting bacterial (PGPB) members of the Rhizobiales and Burkholderiales (including several *Ensifer* as well as *Rhizobium*, *Bradyrhizobium*, and *Rhizobacter*). Besides *Ensifer* (OTU1), only one other OTU was found in 100% of sampled nodules—OTU4, best identified as *Shewanella* (Order Alteromonadales). Our *β*_RC_ analyses of community similarity suggest that nodule communities were much more similar to, and likely deterministically derived from, root communities (70% significant *β*_RC_ values between paired samples; all significant values of *β*_RC_ were < − 0.95, indicating deterministic establishment) rather than rhizosphere communities (26.6% significant *β*_RC_ values) from the same plant (Fisher exact test, *P* = 0.0017), and that this difference persisted independent of genotype and soil origin (all *P* > 0.1). Additionally, we examined if nodule assembly was driven by OTU competition and co-occurrence dynamics. There were no significant co-occurrence patterns for nodule communities (*Z* = 0.304, *P* > 0.05 and *Z* = 0.478, *P* > 0.05 for the entire nodule community and while excluding *Ensifer* respectively) suggesting that these nodule communities do not follow deterministic assembly rules. However, investigation of OTU co-associations with OTU1 (*Ensifer*) resulted in 87 OTUs with significant correlations (26 negative and 61 positive correlations). Interestingly, of our core nodule OTUs, four OTUs were correlated with OTU1; among these, OTU26 (*Rhizobium*) and OTU30 (*Bradyrhizobium*) were significantly negatively correlated with OTU1 (correlation coefficients of − 0.359 and − 0.404 and *P* = 0.024, and *P* = 0.0 respectively), suggesting that these other diazotrophic taxa are competitively excluded by *Ensifer*. The core nodule OTUs, OTU34 (*Halomonas*) and OTU 371 (*Ensifer*), were positively associated with OTU1 (correlation coefficients of 0.390 and 0.593 and P = 0.022, and P = 0.0 respectively).

Despite a strong effect of host genotype on root communities (Table 2), our LEfSe analysis found no OTUs as biomarkers for particular plant genotypes, indicating that these community differences were largely driven by shifting OTU abundance ratios rather than the exclusion of specific taxa. Interestingly, however, when we compared the dissimilarity between root and rhizosphere bacterial communities across the three plant genotypes, root and rhizosphere communities were more alike in plant genotype G96 compared to G1 (Fig. 2b), potentially suggesting that this host genotype G96 is a less stringent “filter” of external bacteria.

### Rhizobium genotype experiment

In stark contrast to the plant genotype × soil experiment, the rhizobium genotype experiment did not yield any differences in diversity estimates across rhizobium strains (*P* > 0.30 for strain for richness, diversity, and evenness) or rhizobium strain × compartment interactions (*P* > 0.4 for all estimators; Table S4). Compartments did differ, however, with the rhizosphere having higher richness (*P* < 0.001), diversity (*P* = 0.002), and evenness (*P* = 0.021) in ANOVA analyses (Table S4). Further, rhizobium strain did not impact communities in PerMANOVA tests for the rhizosphere (*F*_3,14_ = 1.08, *P* = 0.23), root (*F*_3,15_ = 0.97, *P* = 0.48), or leaf endosphere (*F*_3,11_ = 0.94, *P* = 0.57).

### Minimum entropy decomposition of Ensifer (OTU1)

Of the four demarcated minimum entropy decomposition (MED) nodes within *Ensifer* (Table S5), representing within-OTU variation, only two were common (node 3 and node 6; comprising 84.89% and 15.02% of total node counts, respectively). The proportions of these two MED nodes found in plants differed between France and Corsica (*χ*^2^ = 3775.6, *P* < 0.001). Plants grown in Corsican soils had higher node 3 occurrences (87.5% vs. 79.9% in French soil), and reduced node 6 occurrence (12.4% vs. 20.6% in French soil), and these differences were consistent across each of the four plant compartments (Leaf: *χ*^2^ = 2601.9, *P* = 0.001; Nodule: *χ*^2^ = 5647, *P* < 0.001; Root: *χ*^2^ = 16842, *P* < 0.001; Rhizosphere: *χ*^2^ = 12460, *P* < 0.001). Interestingly, *Medicago* genotype affected the proportion of *Ensifer* MED nodes in each compartment (Leaf: *χ*^2^ = 3591.9, *P* < 0.001; Nodule: *χ*^2^ = 4412, *P* < 0.001; Root: *χ*^2^ = 8958, *P* < 0.001; Rhizosphere: *χ*^2^ = 5994.7, *P* < 0.001), suggesting that plant genotypes were differentially colonized by *Ensifer* variants; however, the pattern differed across compartments. Plant genotype G96 contained a smaller proportion of *Ensifer* MED node 3 in nodules and roots, relative to the two other host genotypes (nodules—78.5% in G96 vs. 90.2% and 90.3% in G1 and G27, respectively; root—60.1% in G96 vs. 99.8% and 87.1% in G1 and G27, respectively), but actually had more node 3 in rhizosphere samples (99.9% in G96 vs. 65.2% and 64.9% in G1 and G27, respectively). Within leaves, however, G27 and G96 were similar (> 99% MED node 3), while G1 contained a smaller proportion of node 3 (83.3% node 3, 16.6% node 6). This indicates that *Ensifer* MED node membership within plants is driven by soil origin and host genotype, but patterns are generally consistent independent of plant compartments.

### Metagenomic sequencing

**Fig. 4:**
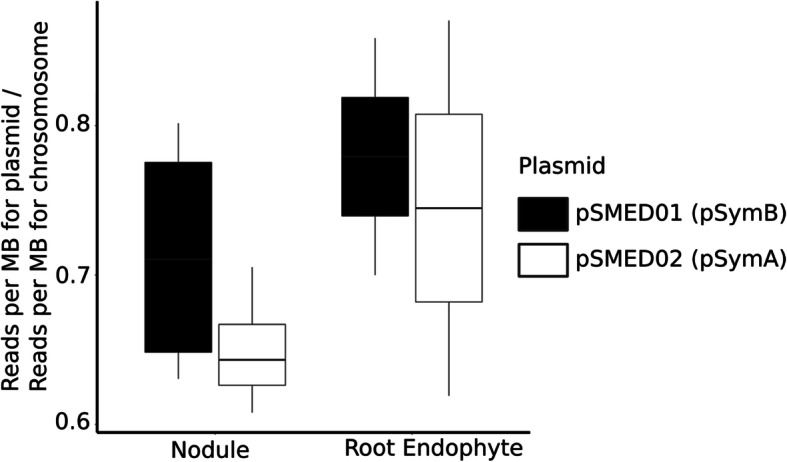
Genomic content of Ensifer from root versus nodule endosphere compartments from shotgun metagenomic sequencing data, shown as the median proportion of reads mapping to the symbiotic plasmid (pSymB in black, pSymA in white) relative to the chromosome. Lower and upper bounds of each box depict the first and third quartiles, respectively, with whiskers representing the range of observed values

Because *Ensifer* was the dominant taxon in all compartments, and MED analysis at the 16S rRNA gene indicated minimal diversity within *Ensifer* OTUs (see above), we used metagenomic shotgun sequencing to further examine genome-wide similarity of *Ensifer* populations across different compartments. *Ensifer* scaffolds reconstructed from root endophyte and nodule communities had, on average, 99% global sequence identity in homologous regions—suggesting that *Ensifer* are extremely similar throughout an individual plant. Moreover, our estimate of the fraction of *Ensifer* cells containing each of the two symbiotic megaplasmids did not significantly differ between plant compartments (*W* = 132, *P* = 0.10 for pSMED01; *W* = 151, *P* = 0.14 for pSMED02; Fig. 4)—again suggesting that *Ensifer* are highly similar throughout the plant, including at the symbiosis megaplasmids.

## Discussion

An increasing number of studies characterize plant microbiomes, moving us toward a more mechanistic and synergistic understanding of factors structuring these communities. Nevertheless, while many studies have looked at spatial variation or plant genetic variation, most studies do not simultaneously examine both soil origin and plant genotype in the same design, making direct comparisons among these effects difficult (but see [33, 62–64]). Our design, combined with sequencing microbial communities from both endosphere (root, nodule, leaf) and rhizosphere compartments, allows us to directly compare the effects of soil and genotype across these distinct “organs.” Here we show that both soil origin and plant genotype contribute to microbiome composition, but that the strength of these effects depend on the compartment—whether the microbes are inside (root endosphere) or outside (rhizosphere) plant tissues. Namely, plant genotype had much stronger effects on microbes within root tissues, while soil origin had stronger effects in the rhizosphere communities. Other interesting findings include the following: (1) that our results mirror those of recent studies in rice [33], poplar [62], and soybean [63] identifying a similar magnitude of effects and a larger role of soil origin than plant genotype in structuring microbiome variation, particularly in the rhizosphere, (2) nodule microbiomes, while containing more than the rhizobium that fixes nitrogen in *Medicago* nodules (*Ensifer*), were much less diverse than the rest of the root endosphere and do not appear to harbor specialist microbial taxa, and (3) *Ensifer* was the dominant OTU, not only in the nodules, but throughout the entire plant. *Medicago* is a well-studied genetic model for plant-microbe symbiosis [60, 61, 65], and ours is the first NGS study of its native soil microbiome; therefore, we anticipate that our results will be of interest to many in the plant genetics community who are interested in building on our mechanistic understanding of 2-player plant-microbe interactions to better understand plant microbiomes. We discuss the major implications of our main results below.

### Soil origin influences the microbiome

Soil microbial communities are remarkably diverse [66] and serve as a source reservoir for plant colonization. Throughout the range of a plant species, soil communities can vary considerably [67, 68], which could potentially confer variation in microbiomes. Here we find that soil origin had a larger effect than plant genotype overall and that rhizosphere communities, in particular, responded strongly to soil source. Finding significant soil origin variation suggests that we would discover additional rhizosphere taxa if we were to sample from more locations (though less so for internalized plant microbiomes, i.e., root, nodule, leaf endospheres). This is noteworthy, as rhizosphere communities are much more diverse than internalized plant communities, and rhizosphere dynamics can have profound impacts on plant fitness (reviewed by [1, 69]). Microbes are recruited to the rhizosphere by plant exudate production (reviewed by [70]). These exudates can provide a nutrient source and interact with edaphic conditions to generate a distinct environment from the surrounding soil that facilitates microbial growth, setting the stage for myriad microbe-microbe interactions that shape this dynamic community [1, 71]. These factors, which structure the rhizosphere, depend heavily on the abiotic and biotic facets of the soil; thus, it is not surprising that soil variation impacts the rhizosphere microbiome. Despite this variation in rhizosphere communities, and the fact that the rhizosphere encapsulates the roots and is the source community for endosphere colonization, internalized plant microbiomes were consistent across soils, consistent with plant genetic, cellular, and/or biochemical mechanisms that restrict entry inside plant tissues (reviewed by [1, 16]).

### Plant genotype structures the Medicago microbiome

Plant genetic variation played a role in structuring variation in the root endosphere community, but not in other compartments. Although we did not find that plant genotype structured rhizosphere communities, studies in other systems have identified such an effect [33, 63]. As mentioned above, root exudates mediate the rhizosphere community assembly, and these exudates are genetically determined [71]. Examination of additional *Medicago* genotypes may very well reveal genetic variation for exudates which may confer rhizosphere variation, and/or nodule and leaf compartments. Importantly, growing plants in closed, bottom-watered boxes likely caused us to underestimate leaf endosphere diversity and miss key taxa; our leaf bacteria likely colonized through vertical migration via plant vasculature, whereas leaves in nature are often colonized from external sources [36, 37].

Plants in our experiment were grown in soils from the native range [58, 72]; therefore, we are likely capturing an ecologically relevant and co-evolving set of microbes that colonize *Medicago* in nature. In particular, the root endosphere taxa that we sampled likely contain many of the “core” players within native *Medicago* plants, at least at the taxonomic scale sampled here (genus or above, see “Discussion” below), because our results join many other studies showing that plants are robust filters of their environmental microbes [1, 73, 74], with soil origin having little effect on internal compartments. Indeed, in our study, microbiome diversity decreased moving from outside to inside the plant—from the rhizosphere to the root/leaf endosphere to the nodule endosphere. This filtering, operating at the boundary between the rhizosphere and the internal tissues, is likely the result of multiple selective processes [1], and our data join others suggesting that at least some of this filtering is plant genotype-dependent (see below).

Our data hint at the existence of quantitative genetic variation for niche breadth in plant microbiomes. We found that the similarity between the endosphere and rhizosphere varied among *Medicago* genotypes, suggesting that some genotypes might represent weaker filters than others. There is empirical evidence for variation along the specialist-generalist continuum within plant-microbe symbioses [75, 76], as well as among plant species, with potential applied implications for the spread of invasive legumes [77, 78]. Using five genotypes of the plant *Boechera stricta*, Wagner et al. [29] found significant genetic variation for metrics of microbiome diversity. Thus while considering such variation in the broader context of niche breadth theory can help us to make sense of plant-microbe symbiosis [76], our ability to interrogate the plant genes controlling microbiome diversity alongside those controlling microbiome composition grows as plant quantitative genetics and microbiome studies come together [16].

The variation in microbial community composition that can be partitioned among plant genotypes, and thus attributed to plant genetic variation, represents the natural variation upon which selection can act in nature and also the amount of standing genetic variation available to plant breeders interested in optimizing plant-microbe interactions. Although the main effect of plant genotype was small (~ 1.3% of total variation), the plant genotype × compartment interaction accounted for much more variation (~ 6.3%) because the effect of genotype was strong, but limited to the root endosphere compartment. These patterns generally mirror studies in other systems (e.g., [33, 62, 79, 80]). The mechanisms by which plant genotype influences the microbiome are still being elucidated, but genetic studies to date suggest that plant genes related to disease resistance, cell walls, and root hair structure may contribute [81–84].

The amount of variation explained by genotype in our experiment is likely an underestimate for multiple reasons. First, we only surveyed three plant genotypes; thus, we cannot account for any genetic variants not represented in these three genotypes. Second, our 16S survey represents species- or even genus-level variation; the long history of plant-microbe symbiosis literature has shown enormous within-species genotypic variation and genotype-by-genotype interactions for infection rates and abundance [85–89]; surely, this variation is also present within at least some of the taxa in the less-studied members of the plant microbiome. Indeed a recent study showed that plant genotype-dependent shifts after multiple serial passages occurred at fine taxonomic scales among closely related OTUs [90]. We have little ability to incorporate these finer-scale genotypic effects using 16S surveys of community composition, though shotgun metagenomic methods for simultaneously addressing population genetics alongside community shifts are quickly evolving (e.g., [91, 92]).

### The nodule microbiome

As expected, the nodule microbiome was dominated by *Ensifer* and was also inhabited by a diverse community, albeit less diverse than the surrounding root endosphere and rhizosphere (Fig. 1). Despite evidence that nodule communities were deterministically sampled from the root endosphere, we did not find evidence that the nodule harbors unique microbial specialists, as no OTUs were found to be biomarkers for the nodule, besides *Ensifer* (Table S3). Nevertheless, we did identify multiple core nodule OTUs that were abundant throughout all nodules. The occurrence of *Shewanella* spp. within all nodules and at great abundance is intriguing, but further experimentation is needed to investigate the potential functional roles of this taxon. We also identified a positive co-association between *Ensifer* and *Halomonas*. *Halomonas* is a moderate halophile and has been demonstrated to improve alfalfa yield (*Medicago sativa*) when co-inoculated with *Ensifer* in saline soils [93]. This suggests the strong potential for *Halomonas-Ensifer* syntrophy within nodules.

Tkazc et al. [80] also identified numerous taxa coinhabiting *Medicago* nodules, but only two OTUs with appreciable sequence counts (greater than 100), *Solirubrobacter* sp., and an *Azohydromonas* sp. While neither of the genera were represented in our core nodule taxa list (Table S4), we did resolve four *Solirubrobacter* OTUs and seven *Azohydromonas* OTUs that were present in nodules (Table S3), but with relatively little sequence representation. These differences are likely attributed to different soil sources; here, we utilized native soils collected from the base of *Medicago* plants in the field, whereas Tkazc et al. [80] used non-native soils where *Medicago* was not present. More research is required to resolve consistent patterns of nodule endophyte associations and how they vary across native and non-native soils, and thus whether taxa like *Azohydromonas*, *Solirubrobacter*, and *Halomonas* have a major functional role in *Medicago* nodules.

Nodules seemingly represent a distinct environment from the adjacent root endosphere, yet many OTUs are found within nodules and across other compartments. Along these lines, there are numerous examples of non-classic rhizobial species (e.g., *Pseudomonas* spp*.*, *Agrobacterium* spp., etc.) that possess nodulation genes, nitrogen fixation genes, or both, being cultured from nodules, suggesting that these microbes may have a specialized role in the nodule [56]. Nevertheless, culturing efforts have identified non-rhizobium species that act to increase nodulation [57]. Additional unidentified, synergistic microbes of this type likely exist, but our findings suggest they will not be strictly restricted to nodules and could be cultured from root or rhizosphere communities.

### Ensifer—a major actor in the Medicago microbiome

Beyond the nodule, our results suggest an extremely dominant role for *Ensifer* throughout the *Medicago* microbiome, both inside and outside the plant. Species in Rhizobiales have been found widely in plant microbiomes, including root and leaf tissues, and from a broad diversity of plants beyond legumes (e.g., [28, 79, 80, 94, 95]). Indeed, we have identified *Rhizobium* and *Bradyrhizobium* in our study, and these taxa are major members of microbiome communities in multiple compartments (Table S2), yet they do not nodulate *Medicago*. Recent phylogenetic reconstructions [96] and mutant screens [97] suggest that such less-specific plant associations predate the origin of root nodule symbiosis in this group. Thus nodulating rhizobia may have evolved from commensal ancestors of plant microbiomes. In many rhizobia, including *Ensifer*, the majority of the genes governing nodulation are contained on symbiosis plasmids [98, 99], and symbiosis genes or entire plasmids can be lost as rhizobia evolve to a commensal lifestyle [96], though this might be unlikely, at least for pSymB, which is currently considered to be a chromid (rather than conjugative plasmid) and contains at least one essential gene [99].

These past observations raised the question of whether the *Ensifer* OTUs in the nodule were distinct from those in other plant compartments, in terms of sequence similarity as well as genome content. Further interrogation of a subset of communities using metagenomic shotgun sequencing suggested that leaf and root endosphere *Ensifer* likely retain their symbiosis plasmids and thus the ability to form nodules and fix nitrogen in the future. Beyond its presence outside the nodules, however, the dominance of *Ensifer* was striking—reaching more than 50% OTU relative abundance (Fig. 1b) even in the leaf tissue. While microbiome studies routinely identify various rhizobium taxa, this level of prevalence among all compartments is unique. Tkazc et al. [80] also identified *Ensifer* as a major component of the microbiome community outside of the nodule; however, it was not the dominant taxon as in our study. Once again this highlights the potential novelty of examining native soil communities. Our soils were collected from the base of the *Medicago* plants and thus *Ensifer* populations were likely enriched through plant-soil feedbacks [100] in the starting soil community. Furthermore in the absence of *Medicago* plants, as was the case for Tkazc et al. [80], the *Ensifer* populations may have adapted to free-living conditions and are not as well suited for plant colonization. Indeed, one hypothesis for the prevalence of *Ensifer* is that they are particularly able to colonize and proliferate in host tissues due to long-standing beneficial symbiosis with *Medicago* species [101], the result of a coevolutionary process within this group and throughout the legume phylogeny, and one that appears to have led to differentiation of signaling interactions among taxa while maximizing the signaling recognition within taxa [102]. The ability to compete for and colonize root nodules in rhizobia is conferred by genes that act in a complex cascade of molecular “handshakes,” including nod factor, exopolysaccharides, and effectors (e.g., [88]; reviewed by [46, 103]). It is possible that these molecules are used throughout the plant tissues to signal entry and allow *Ensifer* to proliferate in all compartments; this hypothesis could be tested by competing nod+ and nod− strains of *Ensifer* (or other nodulation mutants) and testing their relative abundance across host compartments.

In our experiment, we did not find evidence that rhizobium genotype structured microbiome variation across endosphere compartments, or in the rhizosphere, although this question deserves further investigation. Abundant evidence exists demonstrating genetic variation for partner quality (i.e., the fitness benefits that the plant receives from interaction with a given rhizobium strain) among rhizobium genotypes [86, 87, 104, 105]. Given the abundance of rhizobia within plant microbiomes (beyond the nodule), it stands to reason that they could play a pivotal role in microbe-microbe interactions and thus influence plant fitness. Indeed, nodulation mutant plants have been demonstrated to elicit distinctive shifts in microbiome composition [51]. Here we inoculated plants with individual strains of rhizobia, which is a common practice for agriculture and restoration [57]. In future experiments, one could investigate the role of rhizobium genetic variation by inoculating plants with strains that are known to be of high vs. low partner quality, or manipulate strain identity for plants with highly specific strain preference (i.e., partner choice, which is known to vary in *M. truncatula* [106]).

## Conclusions

Here we use a manipulation in native soil to show that plant genetics and soil origin structure different compartments of the *Medicago* microbiome. We also found that *Ensifer* bacteria were abundant throughout plant tissues, where they retain symbiosis plasmids, though we do not yet know whether these symbionts are mutualistic outside the nodule. Future efforts should examine the functional roles and fitness effects of rhizobia in plant microbiomes, both above and belowground. Genetic mapping studies can uncover whether well-known legume symbiosis genes are pleiotropic, affecting interactions with *Ensifer* and other microbes residing throughout the plant. In particular, identifying the genetic basis of root exudate variation may be insightful for understanding mechanisms structuring plant microbiome variation. Leveraging plant genetics and plant breeding to improve plant health via the microbiome is a critical next step, given evidence that controlling microbial colonization through even intensive management and inoculation methods can be challenging in some conditions [107]. Finally, in this and other host-microbiome systems, integrating the vast functional variation known to exist at the strain level (genotypic variation and G × G interactions; reviewed by [89, 108]) with microbial ecology has the potential to reveal much of the hidden heritability of the microbiome.

## Methods

### Overview

To study how host and symbiont genetic variation mediate the microbial communities in the rhizosphere, root endosphere, nodules, and phyllosphere of *Medicago*, we performed two experiments. In the “plant genotype × soil” experiment, we grew three plant genotypes in each of two soils sourced from the native range of *Medicago* to ask how the plant microbiome is structured based on plant genetic variation and what role soil origin contributed to microbiome variation. In the “rhizobium genotype” experiment, we used a single plant genotype and soil community and inoculated with one of four strains of *Ensifer* from two species (2 strains *E. meliloti* and 2 strains of *E. medicae*) to ask if plant microbiomes can be altered by rhizobium genetic variation.

### Plant genotypes, rhizobium strains, and soil sources

Here we chose 3 *Medicago* genotypes (G1, G27, G96) which originated from native populations and are representative of typical *Medicago* plants. While these genotypes were not selected due to *a priori* phenotypic differentiation, our previous work indicates that they possess genetic variation for symbiosis-related genes and phenotypes. These genotypes are differentiated at DMI1, a key symbiosis gene known to be under selection in nature [109, 110]. Additionally, in a previous experiment, these genotypes were inoculated with a mix of 3 rhizobium strains from the native range. In this experiment G96 had marginally higher nodule numbers than G1 (*p* = 0.075) and G27 (*p* = 0.085), as well as larger shoot biomass than G27 (*p* = 0.001). The plant genotype experiment used these three *Medicago* genotypes, whereas only genotype (G96) was used for the rhizobium genotype experiment. Native soils collected from representative wild *Medicago* populations from mainland France (43° 08.845 N, 003° 00.047 E) and from the island of Corsica (42° 58.471 N, 009° 21.861 E) were used in these experiments. Soil was collected from the base of plants growing in the field. Strains of *Ensifer* used in the rhizobium genotype experiment were acquired from culture collections at the University of Minnesota.

### Planting, inoculation, and harvest

Seeds were provided by the Institut National de la Recherche Agronomique (INRA) collection maintained at Station de Génétique et Amélioration des Plantes, INRA, Montpellier, France. For each soil treatment, native soil from either France or Corsica was added in a 1:1 ratio of field soil to sterile root wash soil media (autoclaved at 121 °C four times for 45 min, alternating wet and dry cycles). Before planting, seeds were surface sterilized in dilute (5%) bleach for 5 min, rinsed in sterile water, and cold stratified on moist filter paper for 2 days until seeds germinated. Seedlings were planted with native soil mixture into sterile, sealed, fully self-contained Magenta vessel “leonard jars” to prevent cross-contamination and colonization of microbes other than those found in the native soils [87, 111]. Here 3 GA7 vessels (Caisson Labs, Smithfield UT) were assembled with the bottom jar serving as a water basin, the middle jar filled with soil medium, and the upper jar serving as an empty head space for plant growth. A sterilized nylon wick connected the lower and middle box through a drilled hole. Magenta pots were randomly placed in a temperature-controlled grow room (23 ° C) under artificial light set to 12-h days and randomly rearranged twice per week until harvest.

For the rhizobium genotype experiment, we inoculated three replicate plants (only G96 with only soil from mainland France) grown as above with four rhizobium strains: two *Ensifer medicae* (KH36b and A321) and two *Ensifer meliloti* (M156 and HM007-10). Strains were grown in TY media at 30 ° C and equilibrated to OD_600_ = 0.5 before pipetting 1 ml directly on the soil at the base of the seedlings. Once inoculated, magenta pots were rearranged twice a week and grown concurrently and with the same conditions as the plant genotype × soil experiment. Plants received a second inoculation as above at 16 days after planting.

Plants were harvested after 7 weeks of growth. Magenta vessels were opened in a containment flow hood and plants were excised from the soil. Plants were cut at the root-shoot interface and six randomly selected leaves per plant were placed into a sterile microcentrifuge tube. Leaf tissues were washed with 1% Triton-X 100 (*v*:*v)* by vortexing and rinsing three times with ddH2O to remove any surface particles or epiphytic microbes to ensure only true endophytic microbial members remain [112]. Root tissue was rinsed in ddH_2_O to remove loosely adhered soils. Roots were placed into sterile 50-mL Falcon tubes filled with 40 mL ddH_2_O and agitated thoroughly to remove rhizospheric soils. Next 4 mL of this rhizospheric soil slurry was placed into microcentrifuge tubes and pelleted [113]. These rhizospheric soils were placed directly into PowerSoil DNA Isolation Kit extraction tubes (MoBio; Carlsbad, CA, USA). Washed plant roots were placed into sterile petri dishes and all nodules were harvested and five random living nodules were placed into DNA extraction tubes. Remaining root material was cut into approximately 5-cm sections and six random root segments were collected (a total 30 cm of root tissue), and was placed a microcentrifuge tube, surface sterilized in 30% bleach for 60 seconds, and rinsed in ddH2O three times. Phyllosphere (leaf), rhizosphere, nodule, and endosphere (root) tissues were placed into extraction kits (as above) and stored at − 20 ° C until DNA extraction. Plants in the rhizobium genotype experiment were harvested as above.

### DNA extraction, amplification, and sequencing

Full methods may be found in Supplemental Text S1. In brief, following extraction, bacterial communities were targeted by amplifying the V4 region of the 16S rRNA gene operon as previously described [114] (Supplemental Text S1; Table S6). Samples were pooled, ligated with Illumina-specific sequencing linkers, and sequenced in one reaction of Illumina MiSeq (300PE) at the W. M Keck Center (Urbana IL, USA). Sequence data were processed as previously described using the program mothur (v.1.39.5 [115];), with modifications (see [114, 116, 117]).

### Diversity, communities, and statistical analyses

Observed OTU richness (*S*_obs_), complement of Simpson’s Diversity (1-D), and Simpson’s Evenness (*E*_D_) were estimated (1000 iterations) by subsampling 1500 sequences per sample and the average of these estimators used for downstream analysis. We used the OTU × sample table (Table S1) to generate a pairwise dissimilarity matrix (Bray-Curtis) with the above subsampling and iteration framework. We used fully factorial three-way ANOVA to test for effects of plant compartment, soil origin, or plant genotype on diversity estimators. Data were transformed to increase normality by using logit transformations (1-D and *E*_D_) and Box-Cox transformations (richness) by compartment prior to analysis (Rhizosphere *λ* = 1.6, Root *λ* = 0, Leaf *λ* = − 0.2, Nodule *λ* = − 0.2). To investigate whether bacterial communities shifted across genotypes and soil origin, we conducted a series of one-way PerMANOVAs [118] separately for each compartment (adonis function in the R package *vegan;* R core team 2017 [119];), since initial analysis suggested that communities across plant compartment were extremely different (*F*_3,115_ = 6.32, *P* < 0.001).

We visualized communities using nonmetric multidimensional scaling (NMDS) as implemented in mothur using 1000 iterations (3D stress = 0.123), and used axes loading scores to examine community shifts across genotypes and soil origin where PerMANOVAs were significant. To quantify the amount of bacterial community variation accounted for by each treatment and interaction, we used a modified principal variance component analysis (PVCA [120, 121];). Briefly loading scores from each of the first 10 NMDS axes (representing over > 99% variation) were used as dependent variables in random effects models using restricted maximum likelihood (REML) to determine the percentage of variation explained by each treatment, interactions, and residuals. These variance partitions were then weighted by the percentage of community variation explained by the NMDS axis (*R*^2^) (see [122]) then scaled and summed across all tested axes to total 100%.

We identified biomarker OTUs that were over-represented between plant compartments, soil origins, or for genotypes within compartments using independent linear discriminant analysis effect size (LEfSe [123];) for each comparison. LEfSe uses Kruskal-Wallis and pairwise Wilcoxon signed-rank tests, signed linear discriminant analysis (LDA) log scores, and associated *p* values to identify OTUs that are biomarkers for a particular treatment. Since individual plants were isolated in Magenta boxes, we also used a paired effect size approach on the 20 most abundant OTUs to test whether these taxa differed in abundance across compartments *within* individual plants. To calculate paired effects sizes, the differences between the relative abundance of each OTU for each compartment (within the same plant) were divided by the sum of the relative abundances. We then visualized the obtained effect size quartiles and used paired Wilcoxon signed-rank tests of whether OTUs differed between compartments within a plant.

To investigate the composition of nodule communities, we identified core bacterial taxa within nodules (defined as found in greater than 50% of all nodule samples) to distinguish between transient taxa that may incidentally be found in the nodules from those that may have tighter associations. To test whether nodule communities were deterministically, versus independently stochastically, structured from root or rhizosphere communities, we calculated pairwise *β*_RC_ (Beta Raup-Crick) values [124, 125] to estimate the probability of pairwise community dissimilarity compared to null models based on data randomization. Using the program PaST (v3.12 [126];) with 1000 replicates, we calculated pairwise standardized *β*_RC_ values between nodule and root (or rhizosphere) communities for the same plant in a paired design as above (− 1 ≤ *β*_RC_ ≤ 1, where |*β*_RC_| > 0.95 indicates divergence from null expectation; two-tailed test, alpha = 0.05). We then used Fisher’s exact two-tailed tests on the number of significant paired *β*_RC_ values to determine whether community similarity between compartments differed between genotypes or soil origins, and whether root-nodule and rhizosphere-nodule deterministic assembly rates differed.

To further investigate root nodule colonization dynamics, we investigated if nodule assembly could be explained by OTU competition, suggesting assembly rules driven by co-occurrence dynamics [127]. To do so, we tested our obtained Nodule OTU × sample matrices (as implemented in mothur, both with and without the exclusion of OTU1—*Ensifer*) against a null distribution (using the metric COMBO) [128] with the null model algorithm SIM6—these were selected as these best fit assumed dynamics associated with nodule colonization including that probabilities of occurrence within nodules are proportional to richness and following recommendation by [127] with 10,000 iterations. Additionally, to explore nodule OTU co-associations with *Ensifer*, we utilized a SparCC (Sparse Correlations for Componential Data) [129] following data filtering recommendations [130] as implemented in mothur (10 iterations with 1000 permutations).

### Further interrogating endophytic Ensifer

Because *Ensifer* bacteria were exceedingly common throughout all plant compartments (see “Results”), we examined the potential for sub-OTU variation within OTU1 (*Ensifer*) by first harvesting all sequences from OTUs identified as Rhizobaceae using the script *mothur2oligo* (http://deneflab.github.io/MicrobeMiseq), then used minimum entropy decomposition (MED [131];) to demarcate distinct genetic groupings using Shannon entropy of obtained sequences. Representative sequences for the 17 identified nodes were then compared (BLASTn) against the representative sequences of OTU1, and MED nodes matching OTU1 at > 99% identity were retained (Table S5). This identified four *Ensifer* nodes, two common (MED 3, MED 6) and two rare nodes (MED 101, MED 102). Next we tested whether the distributions of MED nodes differed across soil origin, genotype, or plant compartment using a series of Pearson’s chi-squared tests on node contingency tables, implemented in R (function *chisq.test*; *p* values were simulated using 10,000 Monte Carlo replications because some cells including minor nodes have low expected values).

Because we demarcated multiple MED nodes (see below), we aimed to investigate if these different *Ensifer* strains might have dissimilar genomic and plasmid composition as well, potentially indicative of differential nodulating and diazotrophic capabilities. Thus, we further explored the genomic composition of *Ensifer* throughout the plant using shotgun metagenomic sequencing for a subset of libraries used in 16S sequencing (Table S7). Libraries were prepared using the Tecan UltraLow DNA Library construction kit and sequenced on NovaSeq 6000 150 PE, using NovaSeq SP reagent kit (all library construction and sequencing was performed by the Roy J. Carver Biotechnology center at the University of Illinois). We generated, demultiplexed, and adapter-trimmed fastq files using bcl2fastq v2.2 then assessed sequence quality using FastQC and performed quality control using bbduk (qtrim = rl trimq = 12 hdist = 1 k = 27 minlenfraction = 0.6 minlen = 40 maxns = 1 maq = 8) [132]. To remove sequences corresponding to plant DNA, reads were mapped against the *Medicago* Mt 4.0 genome [133] using bowtie2 --sensitive [134]. Read pairs in which either read mapped to the *Medicago* reference were removed.

We used the shotgun data in two ways to examine the similarity between *Ensifer* found outside versus inside the nodule compartment. First, we compared whole-genome similarity of populations of *Ensifer* from different plant compartments of the same plant. We assembled microbial reads using metaSPAdes 3.14.0 [135]. We used sendsketch.sh in the BBtools suite [132] to identify scaffold taxonomic identity, and we compared scaffolds greater than 1 kb in each assembly to RefSeq [136]. Scaffolds that had best hits to any *Ensifer* genome were then selected, and BLASTn [137] (ungapped) was used to determine global sequence identity between *Ensifer* scaffolds across endophyte and nodule compartments from within the same plant (when scaffolds ≥ 100 km and average nucleotide identity ≥ 95% or *Ensifer* in RefSeq). Foliar samples were overwhelmed with *Medicago* sequences, and < 1% of the remaining reads mapped to *E. medicae* WSM419, so these were omitted from further analyses.

Differences in gene content could exist between otherwise similar genomes [138]. Thus, we next asked whether all *Ensifer* had similar genome content (chromosome, pSymA, and pSymB) by comparing the relative abundance of reads mapping to each of the two symbiotic plasmids relative to those mapping to the chromosome. To do this, we first mapped reads to a reference assembly (*E. medicae* WSM419 assembly [139] using Bowtie2 in -sensitive mode), which was the most similar and used FeatureCounts [140] to parse the output of Bowtie2 and determine the number of reads mapped onto each coding sequence. Samples with fewer than 20 reads mapping to common core gene *rpoB* [141] belonging to *Ensifer medicae* WSM419 were not included in further analyses, as the signal to noise ratio of these samples was expected to be low. To ask whether symbiotic plasmid abundance might change across compartments, we calculated an estimate of the fraction of *Ensifer* cells possessing each of pSymA and pSymB. For each of the remaining samples, we divided the reads (per megabase) that mapped to the plasmid by the reads (per megabase) that mapped to the chromosome (representing all *Ensifer* cells), then tested whether this ratio differed between root endosphere and nodule compartments using a Wilcoxon-Mann-Whitney test.

## Supplementary information

**Additional file 1:.** Table S1 Full matrix of sequence counts for OTUs (retained) with sample identification. Listed in sample name are soil location (C – Corsica; F – France), genotype (1, 27, 96), replication number (a, b, c, d, e), and plant compartment (L – Leaf; N – Nodule; R – Root (endosphere); and S – Soil (rhizosphere)).

**Additional file 2:.** Table S2 Full OTU information including total sequence count, sequence distribution across compartments, sequence distribution across genotypes, sequence distribution across soil location, LEfSe results (where significant) and to which compartment, LEfSe results (where significant) and to which soils, representative OTU sequences, and full OTU taxonomy string (with bootstrap support for taxonomic rank).

**Additional file 3:.** Figure S1 Venn diagrams of shared OTUs across compartments (a) and soil sources (b). Only OTUs greater than 10 sequences within a particular compartment are included.

**Additional file 4:.** Figure S2: Nonmetric Multidimensional Scaling plot (Bray-Curtis) of rhizopsheric bacteria plotted by soil origin (France or Corsica). PERMANOVA statistics indicate that communities differ between soil origin. Insert represents Axis 3 loading scores (explains 6.61% of community variation) across soil origin (t-test) showing Corsican samples have lower average loading scores than French soils.

**Additional file 5:.** Table S3 Core OTUs with taxonomy found within nodule communities across sampling design, an OTU was defined as core if present in >50% nodule samples

**Additional file 6: **Table S4 ANOVA results of *Ensifer* Inoculation experiment are presented across compartments, *Ensifer* genotypes, and compartment × genotype interactions. Complement of Simpson’s Diversity and Evenness were transformed using logit transformation and Richness was transformed using Box-Cox functions (λ = 0.478) prior to analysis.

**Additional file 7: **Table S5 Results of BLASTn identification of all demarcated *Ensifer* MED nodes. Where these MED nodes best matched *Ensifer* sp. or our OTU1 representative sequence, full blast results are also presented. The four nodes that matched to OTU1 were used for analysis.

**Additional file 8:.** Supplementary Text S1 Supplementary methods for DNA extraction, amplification, and sequencing, and bioinformatics for 16S rRNA gene sequencing.

**Additional file 9:.** Table S6 Primer and MID sequence information for 16S rRNA gene amplicon sequencing, with sample identifications. MIDs and forward primer (515f) were used for secondary PCR reactions.

**Additional file 10: **Table S7 Sequencing and Assembly Information for Metagenomic Samples. Count of sequences per sample before and after quality control, as well as count of sequences considered plant sequences and *Ensifer* sequences for each sample. Summarization of reads mapped to plasmid and chromosome for *Ensifer medicae* WSM419 for each sample.

## Data Availability

Datasets from 16S rRNA gene and shotgun metagenomic sequencing are archived at the Sequence Read Archives (SRA) at NCBI under the BioProject accession numbers PRJNA663350 and PRJNA663573.
