# Correction to: Soil origin and plant genotype structure distinct microbiome compartments in the model legume *Medicago truncatula*

**DOI:** 10.1186/s40168-021-01080-3

**Published:** 2021-05-10

**Authors:** Shawn P. Brown, Michael A. Grillo, Justin C. Podowski, Katy D. Heath

**Affiliations:** 1 Department of Plant Biology, University of Illinois, 505 S. Goodwin Ave, Urbana, IL 61801 USA; 2 Carl R. Woese Institute for Genomic Biology, University of Illinois, 1206 W. Gregory Dr, Urbana, IL 61801 USA; 3 Department of Biological Sciences, The University of Memphis, 3774 Walker Ave, Memphis, TN 38152 USA; 4 Center for Biodiversity Research, The University of Memphis, 3774 Walker Ave, Memphis, TN 38152 USA; 5 Department of Biology, Loyola University Chicago, 1032 W. Sheridan Rd, Chicago, IL 60618 USA; 6 Department of Geophysical Sciences, University of Chicago, 5734 S Ellis Ave, Chicago, IL 60637 USA

**Correction to: Microbiome 8, 139 (2020)**

**https://doi.org/10.1186/s40168-020-00915-9**

Following publication of the original article [1], an error was identified in the Availability of data and materials section. The updated Availability of data and materials section is given below and the change highlighted in bold typeface.

Datasets from 16S rRNA gene and shotgun metagenomic sequencing are archived at the Sequence Read Archives (SRA) at NCBI under the BioProject accession numbers **PRJNA663550** and PRJNA663573.

The original article [1] has been corrected.
