# Supplementary figures and images for: Soil origin and plant genotype structure distinct microbiome compartments in the model legume Medicago truncatula
Source: Microbiome. 2020 Sep 28;8:139. doi: 10.1186/s40168-020-00915-9 (PMC7523075; doi:10.1186/s40168-020-00915-9)

a)

Nodule

Leaf

Root

Rhizosphere

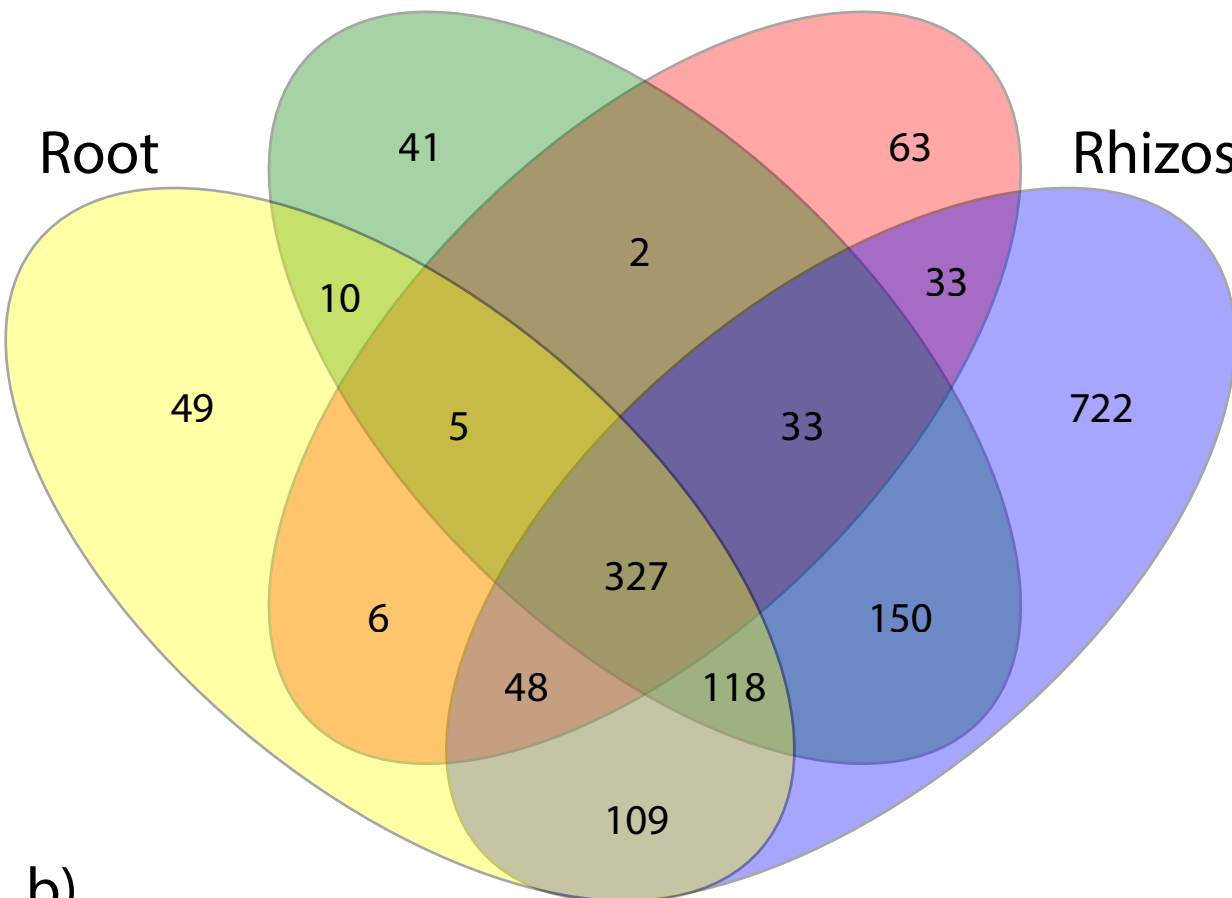

b)

France

Corsica

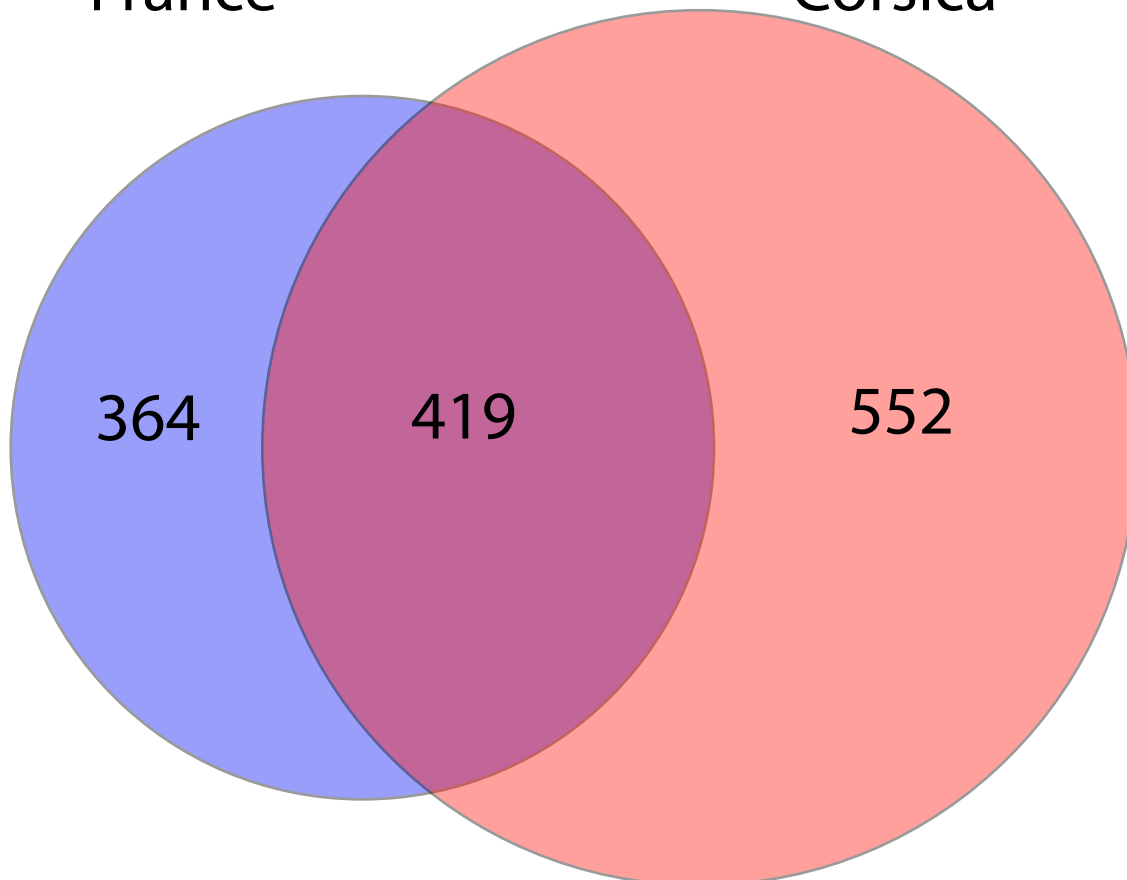

Supplement: Supplementary file 4 — Additional file 3:. Figure S1 Venn diagrams of shared OTUs across compartments (a) and soil sources (b). Only OTUs greater than 10 sequences within a particular compartment are included. [file 40168_2020_915_MOESM3_ESM.pdf]

# NMDS Rhizosphere

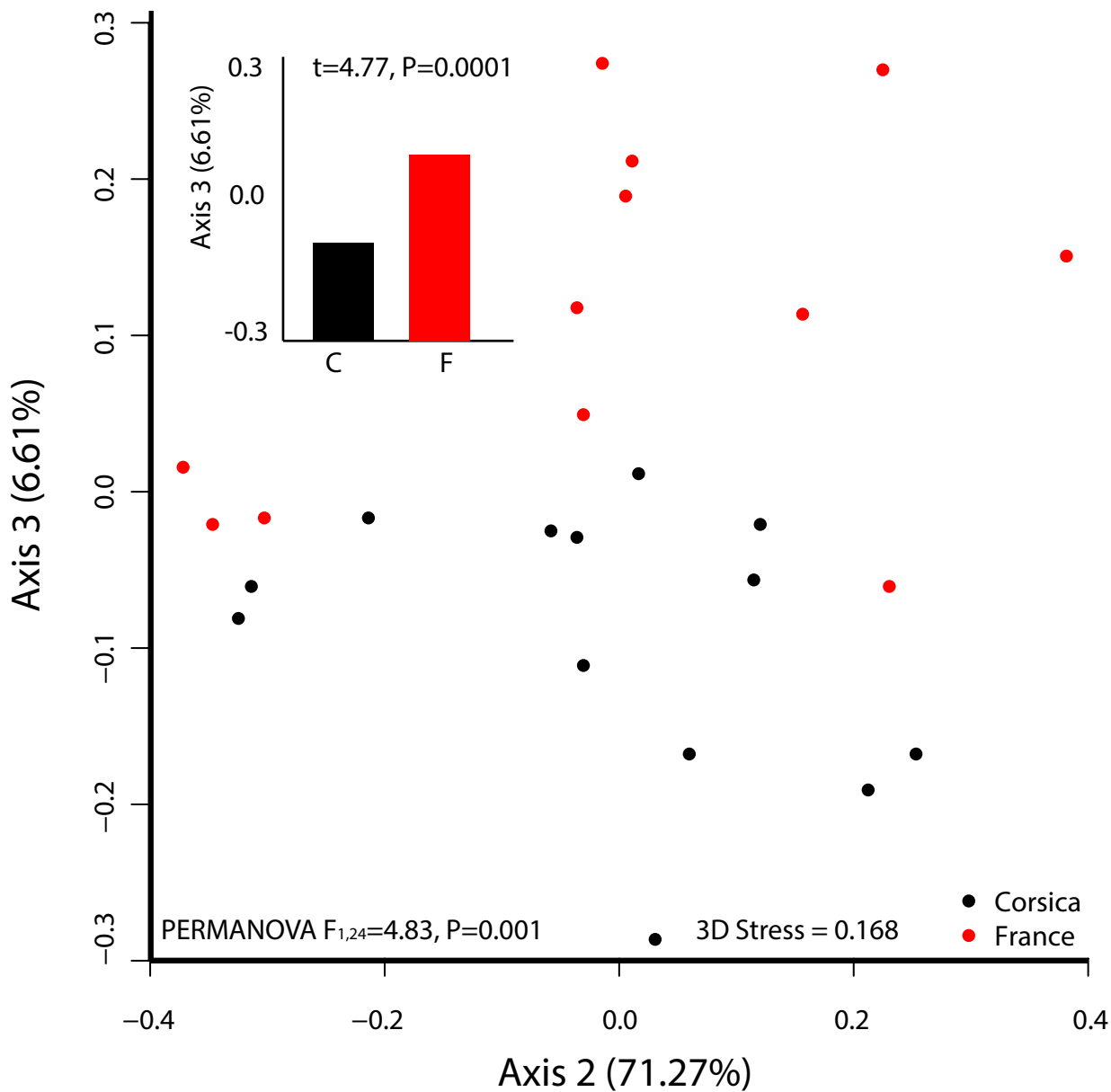

Supplement: Supplementary file 5 — Additional file 4:. Figure S2: Nonmetric Multidimensional Scaling plot (Bray-Curtis) of rhizopsheric bacteria plotted by soil origin (France or Corsica). PERMANOVA statistics indicate that communities differ between soil origin. Insert represents Axis 3 loading scores (explains 6.61% of community variation) across soil origin (t-test) showing Corsican samples have lower average loading scores than French soils. [file 40168_2020_915_MOESM4_ESM.pdf]
