# Supplementary material for: Soil origin and plant genotype structure distinct microbiome compartments in the model legume Medicago truncatula
Source: Microbiome. 2020 Sep 28;8:139. doi: 10.1186/s40168-020-00915-9 (PMC7523075; doi:10.1186/s40168-020-00915-9)
Supplement: Supplementary file 7 — Additional file 6: Table S4 ANOVA results of Ensifer Inoculation experiment are presented across compartments, Ensifer genotypes, and compartment × genotype interactions. Complement of Simpson’s Diversity and Evenness were transformed using logit transformation and Richness was transformed using Box-Cox functions (λ = 0.478) prior to analysis. [file 40168_2020_915_MOESM6_ESM.docx]

**Table S4** ANOVA results of *Ensifer* Inoculation experiment are presented across plant compartments, *Ensifer* genotypes, and compartment x genotype interactions. Complement of Simpson’s Diversity and Evenness were transformed using logit transformation and Richness was transformed using Box-Cox functions (λ = 0.478) prior to analysis.

| Diversity Estimator | Test | F_df_, P-value |
| --- | --- | --- |
| ***Diversity (1-D)*** | ***Plant Compartment*** | ***F_2,43_=7.58, P=0.002*** |
| Diversity (1-D) | *Ensifer* Genotype | F_3,43_=1.16, P=0.339 |
| Diversity (1-D) | Compartment x Genotype | F_6,43_=0.24, P=0.958 |
| ***Evenness (E_D_)*** | ***Plant Compartment*** | ***F_2,43_=4.35, P=0.022*** |
| Evenness (E_D_) | *Ensifer* Genotype | F_3,43_=1.07, P=0.375 |
| Evenness (E_D_) | Compartment x Genotype | F_6,43_=1.02, P=0.429 |
| ***Richness (S_obs_)*** | ***Plant Compartment*** | ***F_2,43_=12.47, P<0.001*** |
| Richness (S_obs_) | *Ensifer* Genotype | F_3,43_=0.85, P=0.477 |
| Richness (S_obs_) | Compartment x Genotype | F_6,43_=0.35, P=0.901 |
