# Supplementary material for: Soil origin and plant genotype structure distinct microbiome compartments in the model legume Medicago truncatula
Source: Microbiome. 2020 Sep 28;8:139. doi: 10.1186/s40168-020-00915-9 (PMC7523075; doi:10.1186/s40168-020-00915-9)
Supplement: Supplementary file 9 — Additional file 8:. Supplementary Text S1 Supplementary methods for DNA extraction, amplification, and sequencing, and bioinformatics for 16S rRNA gene sequencing. [file 40168_2020_915_MOESM8_ESM.pdf]

Supplementary Text S1: Supplementary methods for DNA extraction, amplification, and sequencing

DNA extraction followed manufacturer's protocol with the following modification. Three 2mm zirconia beads were added to the tubes and all samples were ground and homogenized in a Mini-Beadbeater-96 (BioSpec, Bartlesville, OK USA) at 36 oscillations/second for 60 seconds. Extracted DNA was quantified with a NanoDrop 2000c spectrophotometer (Thermo Scientific, Wilmington, DE, USA). Genomic DNA was standardized to a concentration of 20 ng/µl for downstream amplicon library production. Bacterial communities were targeted by amplifying the V4 region of the 16S rRNA gene operon with the primers 515f and 806r [1], with the addition of a unique (per sample) molecular identifier (MID) tag on the 515f primer (following [2]; Table S1). We generated two separate amplicon libraries, facilitating duplicate MID usage.

Amplicons were generated using a two-step procedure in 50µl PCR reactions. Primary PCR conditions consisted of 5µl of both forward and reverse primer (without MID inclusion), 25 µl Phusion High-Fidelity 2x PCR Master mix with HF buffer (New England Biolabs, Ipswich, MA, USA), 5µl of template DNA (100 ng) and molecular grade water for a total reaction volume of 50µl. PCR parameters were: 98C for 30 seconds, 20 cycles of 98C for 10 seconds, 54C for 30 seconds, and 72C for 60 seconds followed by a final extension phase at 72C for 10 minutes. Secondary PCR was as above with the exception of only 2.5µl or forward and reverse primers (with MIDs) used, and DNA template was the primary PCR product, with additional water to make a final reaction volume of 50µl. PCR parameters were as above with the exception of only 10 cycles, for a total amplification of 30 cycles.

To confirm amplification, PCR products (5µl) were visualized on 1% (w:v) agarose gels. Negative controls (sterile water in place of DNA template) were included throughout extraction

and PCRs and remained free of visual amplification. PCR products were cleaned using Agencourt AMPure XP beads (Beckman Coulter, Brea, CA) according to manufacturer protocol with the modification that we used a 1:1 bead to reaction ratio to further discriminate against short fragments [3]. DNA was quantified and then pooled to an equimolar concentration and the combined product was cleaned again with Agencourt AMPure XP beads as above. Illumina specific sequencing linkers were ligated onto the two libraries using the Kapa rapid ligation system with a three cycle PCR clean up step with two separate indexes, pooled, and a final AMPure XP bead cleanup was performed. Final PCR product was then sequenced on one reaction of Illumina MiSeq (300PE) at the W.M Keck Center (Urbana IL, USA).

Sequence data were processed using the program mothur (v.1.39.5; [4] following the MiSeq protocol [5] with modifications. Obtained forward and reverse fastq files were screened for quality using with Q>30 over a 50 bp sliding window as minimum for inclusion. From these, forward and reverse reads from both libraries were contiged and primers were trimmed. Sample specific MIDs (Table S1) were used to parse sequencing into individual experiments (plant genotype x soil and rhizobium genotype) and experimental units and files merged. These experiments were processed and analyzed separately. Sequences were then aligned against a Silva 16S reference alignment (v123) and were screened for off target amplification by assigning each sequence to taxonomic identities (mothur implemented naïve Bayesian classifier; [6]) and all plastid, mitochondrial, and non-bacterial sequences were removed. Sequences were pre clustered to reduce sequencing errors (pseudo single-linkage; [7]) and putative chimeras were identified with the UCHIME [8] and removed. A pairwise sequence distance file (Needleman-Wunsch) was generated and sequences were demarcated into OTUs using the average-neighbor

method in mothur (UPGMA) with a 97% similarity threshold. All OTUs that were found less than 10 times globally were removed to prevent inclusion of potentially spurious OTUs [9,10].

References

1. Caporaso JG, Lauber CL, Walters WA, Berg-Lyons D, Huntley J, Fierer N, et al. Ultra-high-throughput microbial community analysis on the Illumina HiSeq and MiSeq platforms. ISME J. Nature Publishing Group; 2012;6:1621–4.

2. Jones JM, Heath KD, Ferrer A, Brown SP, Canam T, Dalling JW. Wood decomposition in aquatic and terrestrial ecosystems in the tropics: contrasting biotic and abiotic processes. FEMS Microbiol Ecol [Internet]. Oxford Academic; 2019 [cited 2020 Mar 29];95. Available from: https://academic.oup.com/femsec/article/95/1/fiy223/5184448

3. Brown SP, Jumpponen A. Contrasting primary successional trajectories of fungi and bacteria in retreating glacier soils. Molecular Ecology. 2014;23:481–97.

4. Schloss PD, Westcott SL, Ryabin T, Hall JR, Hartmann M, Hollister EB, et al. Introducing mothur: Open-Source, Platform-Independent, Community-Supported Software for Describing and Comparing Microbial Communities. Appl Environ Microbiol. American Society for Microbiology; 2009;75:7537–41.

5. Kozich JJ, Westcott SL, Baxter NT, Highlander SK, Schloss PD. Development of a Dual-Index Sequencing Strategy and Curation Pipeline for Analyzing Amplicon Sequence Data on the MiSeq Illumina Sequencing Platform. Appl Environ Microbiol. American Society for Microbiology; 2013;79:5112–20.

6. Wang Q, Garrity GM, Tiedje JM, Cole JR. Naive Bayesian classifier for rapid assignment of rRNA sequences into the new bacterial taxonomy. Appl Environ Microbiol. 2007;73:5261–7.

7. Huse SM, Welch DM, Morrison HG, Sogin ML. Ironing out the wrinkles in the rare biosphere through improved OTU clustering. Environ Microbiol. 2010;12:1889–98.

8. Edgar RC, Haas BJ, Clemente JC, Quince C, Knight R. UCHIME improves sensitivity and speed of chimera detection. Bioinformatics. 2011;27:2194–200.

9. Brown SP, Veach AM, Rigdon-Huss AR, Grond K, Lickteig SK, Lothamer K, et al. Scraping the bottom of the barrel: are rare high throughput sequences artifacts? Fungal Ecology. 2015;13:221–5.

10. Oliver AK, Brown SP, Callaham MA, Jumpponen A. Polymerase matters: non-proofreading enzymes inflate fungal community richness estimates by up to 15 %. Fungal Ecology. 2015;15:86–9.
